# Responses to salinity stress in bivalves: Evidence of ontogenetic changes in energetic physiology on *Cerastoderma edule*

**DOI:** 10.1038/s41598-018-26706-9

**Published:** 2018-05-29

**Authors:** Laura G. Peteiro, Sarah A. Woodin, David S. Wethey, Damian Costas-Costas, Arantxa Martínez-Casal, Celia Olabarria, Elsa Vázquez

**Affiliations:** 1 Departament of Ecology and Marine Biology, University of Vigo, 36200 Vigo, Spain; 2 Toralla Marine Station (ECIMAT), University of Vigo, 36311 Vigo, Spain; 3 Department of Biological Sciences, University of South Carolina, Columbia, 29208 USA

## Abstract

Estuarine bivalves are especially susceptible to salinity fluctuations. Stage-specific sensibilities may influence the structure and spatial distribution of the populations. Here we investigate differences on the energetic strategy of thread drifters (3–4 mm) and sedentary settlers (9–10 mm) of *Cerastoderma edule* over a wide range of salinities. Several physiological indicators (clearance, respiration and excretion rates, O:N) were measured during acute (2 days) and acclimated responses (7 days of exposure) for both size classes. Our results revealed a common lethal limit for both developmental stages (Salinity 15) but a larger physiological plasticity of thread drifters than sedentary settlers. Acclimation processes in drifters were initiated after 2 days of exposure and they achieved complete acclimation by day 7. Sedentary settlers delay acclimation and at day 7 feeding activity had not resumed and energetic losses through respiration and excretion were higher at the lowest salinity treatment. Different responses facing salinity stress might be related to differences in habitat of each stage. For sedentary settlers which occupy relatively stable niches, energy optimisation include delaying the initiation of the energetically expensive acclimation processes while drifters which occupy less stable environments require a more flexible process which allow them to optimize energy acquisition as fast as possible.

## Introduction

Bivalves are critical to estuarine function in terms of stimulation of microphytobenthic productivity through their release of ammonium and carbon dioxide as well as pelletization of fine particulate matter in addition to their importance as a critical link in energy conversion between primary producers and a variety of predators such as fish, birds, and crabs as well as humans^1^. One of the predicted consequences of global climate change is an increase in the occurrence of extreme climatological events such as droughts or extreme rainfalls that modify coastal salinity^2^. Estuaries, as transitional zones, will experience greatly those salinity fluctuations^3^. For bivalves, salinity is particularly problematic because it drives a reduction in activity and energy acquisition while also increases energy demand for maintaining cell volume and avoiding osmotic shock^4–7^. The maintenance of cell volume is achieved mainly by regulation of the nitrogen metabolism, which also increases oxydative metabolism demands and therefore mobilization of reserves resulting in elevated oxygen consumption rates^4–7^. Given the pairing of this higher metabolic demand with reductions of energy input from food because of reduced feeding activity^4–7^, the net effect of energy trade-offs at the individual level can be severe and translate to mass mortality events^8,9^ or indirectly affect population dynamics by altering growth performance, reproductive output or immune function^4–7,10^.

The cockle *Cerastoderma edule* is one of the most abundant bivalve species in tidal flats where it can comprise 60% of benthos biomass^11^ being critical to ecosystem function in addition to supporting several traditional fisheries^12–15^. This short-lived species is known to have high spatial and temporal variability^10^ but during the last decades cockle stocks have shown a progressive declining trend^12,13,16–19^. Declines of stocks are mostly due to mass mortality episodes and recruitment failures; both provoked by climate-related events^8,9,12,16,17,19–24^. The literature suggests that mass mortality events in cockles are often linked to torrential rains^8,9,17,19,21,22^ with the magnitude of a mortality event depending on the intensity and duration of the flooding episodes maintaining salinity levels below the lethal physiological threshold of the species (10–12.5)^4,5,7,8,25,26^. In fact, an increase in the frequency of flooding events in estuaries has been related to decrements in biomass of *C. edule* and to reductions in the fishery yields^8,19,21,22^. Timing of torrential rains can also be critical. The decrease of recruitment observed in cockles associated with delays on settlement through spring and summer has been related mostly to impairments with food sources (phytoplankton blooms) and seasonal peaks of predator abundance^10,16,20,27^, but sudden drops of salinity during the settlement season could also cause recruitment failure driven perhaps by differing physiological thresholds of early developmental stages. Ontogenetic changes of environmental requirements are common in species, especially for those which occupy distinct habitats during developmental stages^28–37^. A critical aspect of the life history of *C. edule* is that initial settlement occurs in the high intertidal apparently to minimize competition with adults and reduce predation risk^38–40^. After a period of growth the juveniles undergo a post-settlement migration via byssal thread drifting into the lower intertidal where environmental conditions are more stable^41,42^. *C. edule* lose their drifting and climbing abilities when they reach sizes ≈6 mm,which has been associated with the degeneration of the byssal glands^43^. Morphological and habitat differences of thread drifters support their consideration as a distinct developmental stage, which may have specific physiological requirements. Stage-specific requirements may contribute to episodic recruitment failures, as well as the spatial distribution of the species in different estuaries^13,44^. In order to reverse the decline of the stocks, management strategies (delimiting nursery areas, seeding and fishing grounds, etc) need to identify the spatio-temporal availability of suitable habitats for each developmental stage, which will be highly determined by their particular environmental constrictions^13,45^.

This study aims to identify stage-specific salinity requirements related to the loss of drifting capability after settlement and how that affects their physiological response to stress episodes of different duration. With that purpose, we designed a laboratory experiment where thread drifters and sedentary settlers were exposed to a wide range of salinities with reference to the following question while measuring the components of scope for growth (respiration, excretion and clearance rate):

Do the thread drifters and sedentary settlers differ in their response to acute (2 days of exposure) and acclimated (7 days of exposure) response to saline stress? One might expect thread drifters to be less susceptible to acute stress because they need to cope with a highly variable environment at the high-intertidal. On the other hand, one might expect the sedentary settlers to have a better acclimated response because they have lost their migratory capability so they need to develop better acclimated responses.

## Material and Methods

### Experimental setup

Laboratory spawns of adult cockles yielded larvae, which were raised until metamorphosis. Spat were maintained at salinity 35 ± 0.5 for 2–3 months and then divided into two size classes according to their drifting capability (<6 mm)^43^: thread drifters (D; L: 3.74 ± 0.67 mm; n = 224) and sedentary settlers (S; L: 9.66 ± 1.31 mm; n = 84) before transferring to the salinity treatments to simulate a sudden drop in salinity caused by a torrential rain.

Two replicated beakers (1 L) per Salinity treatment (8 levels: 3, 5, 10, 15, 20, 25, 30 and 35), Size (2 levels: D and S) and time of exposure (2 and 7 days) were maintained in an orthogonal design in a controlled temperature room at 14 °C. No sand was included in the beakers since preliminary studies reported no differences in survival or pumping rates with/without sediment in laboratory experiments^25,46^. Density per beaker was adjusted to those determined in preliminary experiments to give replicable results for rate measurements (250 and 45 indiv/beaker for D and S, respectively). Water was changed daily before feeding (1% of dry weight of microalgae per cockle live weight) with a mixture of *Isochrysis galbana* (TISO), *Tetraselmis suecica*, *Chaetoceros gracilis* and *Rodomonas lens* (1:1:1:1).

Physiological performance metrics (respiration, ammonium excretion and clearance rates) were measured on day 2 and 7 of the experiment, considering day 2 as acute stress response and day 7 as acclimated stress response.

### Physiological measurements

Each replicate was divided into two pseudo-replicates to measure physiological performance of the individuals during acute and acclimation response to salinity treatments.

### Clearance Rate

Clearance rate (CR; Lh^−1^) was calculated on pools of cockles (40 and 15 ind. for D and S Size treatments, respectively) in a static system. Two plastic beakers of 100 ml with filtered seawater (50 µm) were used per replicate. A pool of individuals was placed in each beaker and after 30 minutes of acclimation, ≈400000 cells ml^−1^ of *I. galbana* were added while aeration was kept high to maintain suspension of the microalgae. During basal measurements at salinity 35 a sample of 10 ml was taken from the centre of the beaker with a pipette, every 5 minutes during 30 minutes to establish the period of time with maximum CRs for each size class (15 and 10 minutes for D and S, respectively). These periods of time were used to take CR samples throughout the rest of the experiment. Two beakers without cockles, but with the same microalgal density were also sampled at the beginning and at the end of each run of measurements to subtract the difference on particles caused by sedimentation of microalgae to the concentration obtained at the experimental beakers. Samples were fixed with lugol to avoid degradation until processing with a counter coulter (Beckman Coulter Multisizer 3) to calculate particle concentration.

CR was calculated with the following equation^47^:

$${\rm{CR}}={\rm{v}}(\mathrm{ln}\,{{\rm{c}}}_{0}-\,\mathrm{ln}\,{{\rm{c}}}_{1})/{\rm{t}}$$

where v is the volume of seawater in the beaker and c_0_ and c_1_ are the cell concentrations at the beginning and at the end of the time interval (t). CR was also corrected by the number of individuals in the pool.

### Respiration Rate

Oxygen consumption rate (R; mgO_2 _h^−1^) was calculated on pools of cockles (75 and 15 individuals for D and S, respectively) using closed respirometers attached to dissolved oxygen probes (Hach Lange LDO101). Two cylindrical respirometers of 150 ml filled with aerated 50 µm-filtered seawater at 14 °C were used per replicate. A pool of individuals was placed in the respirometer while seawater was carefully moved with a magnetic stirrer. Dissolved oxygen concentration was recorded every 30 seconds until it declined ≈20% from the initial value. R was calculated using the slope of the relationship between oxygen concentration and time elapsed, and corrected by the chamber volume and the number of individuals in the pool. Two empty respirometers were monitored simultaneously with each run to subtract the difference on oxygen concentration caused by electrode drift, bacterial respiration, etc. from the values obtained at the experimental respirometers.

### Ammonium Excretion

Ammonium excretion rate (ER; mg NH_4_-N h^−1^) was calculated on pools of cockles (40 and 5 individuals for D and S, respectively) after CR measurements. One chamber of 20 ml filled with 50 µm filtered seawater was used per replicate. A pool of individuals was placed in the chamber and, after 1.5–2.5 hours, a sample of 10 ml was collected and ammonium concentration calculated by the phenolhypochlorite method^48^. Two empty chambers were used per run as controls to subtract other ammonium sources from the values obtained at the experimental chambers and correct ERs which were also divided by the number of individuals in the pool.

### O:N index

O:N index was calculated as the ratio between oxygen atoms consumed per atom of nitrogen excreted, using their atomic equivalents over the R and ER values previously calculated. O:N represents the balance between carbohydrates and lipids versus protein catabolism. Balanced catabolism would oscillate between 50 and 60^49^ while values below 30–20 are indicative of stress^6,26,49^ and pure protein catabolism will reach values between 3 and 13^49^.

### Standardization of physiological rates

All the physiological rates were standardised to 0.1 g DW_tissue_ (DW_s_ = 0.1) using the formula:

$${{\rm{Y}}}_{{\rm{s}}}={{\rm{Y}}}_{{\rm{ob}}}\,\ast \,{({{\rm{DW}}}_{{\rm{s}}}/{{\rm{DW}}}_{{\rm{ob}}})}^{{\rm{b}}}$$

where Y_s_ is the standardized rate for a selected weight (DW_s_), Y_ob_ is the observed physiological rate for an animal of a particular weight (DW_ob_), and b is the weight exponent for the physiological rate to weight allometry. We employed b = 0.49 for CR and b = 0.77 for R and ER^50^.

Pools of organisms used for the physiological measurements for the sedentary size class were dissected to calculate the tissue dry weight (DW_tissue_). After dissecting the tissue from the shell, both were dried separately at 60 °C for 48 h and then weighed. Because of the small size of the thread drifters, dissections of animals from that age class were made under the microscope only on a subsample of 10 individuals covering a wide range of sizes from each replicate. Tissue and shell were dried separately and weighed to calculate allometric relationships between DW_total_ and DW_tissue_ per replicate using regression models on log-transformed weights. The rest of the pool was dried without dissecting to calculate DW_total_, and regressions calculated per each replicate were employed to extrapolate DW_tissue_ for each pool.

### Data Analysis

Generalized additive models (GAMs), as implemented in the mgcv library of R 3.3.2, were used to investigate the effect of Size and Salinity on the standardized physiological rates (R, CR and ER) and O:N index on day 2 (Acute stress) and day 7 (Acclimation). This analysis type is useful when the form of the response can be complex and it is difficult to pre-judge the various parametric options^51^. The significance of the interaction between Size and Salinity was evaluated by a likelihood-ratio test comparing Akaike Information Criterion of the model with and without interaction. Size was included as a factor (D and S) and Salinity was included as a smoothed term in the model, using thin plate regression splines and estimating for each size level when the interaction was significant. Model validation included the verification of homogeneity (lack of structure of the residuals) and normality (quantile−quantile plot of the residuals)^52^.

All the analysis were performed on R.3.3.2.^53^.

### Data availability

Raw data has been made available through PANGAEA data repository (https://doi.pangaea.de/10.1594/PANGAEA.889440). The authors also compromise to make materials, other data and associated protocols available to the Editorial Board Members of Scientific Reports, referees contacted by the journal or readers without undue qualifications in material transfer agreements.

## Results

### Acute Response

**Figure 1:**
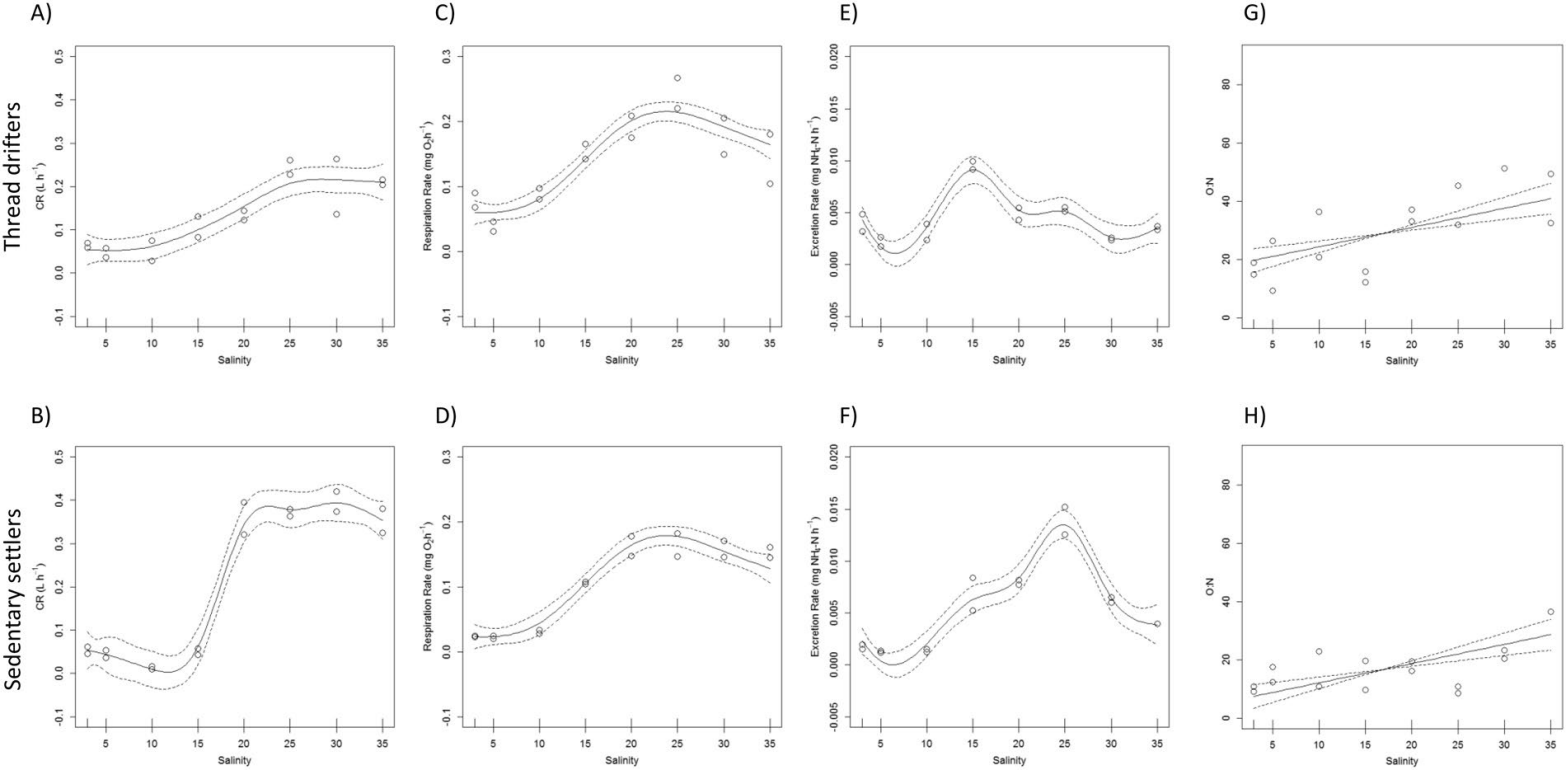
Generalized additive model results showing the partial effect of Salinity on clearance (**A**,**B**) respiration (**C**,**D**) and excretion rates (**E**,**F**) as well as O:N ratios (**G**,**H**) during acute response (2 days of exposure) for thread drifters (top row) and sedentary settlers (bottom row). Dotted lines indicate 95% confidence intervals, and tick marks along the *x*-axis below each curve represent effect values where observations occurred.

After 2 days of exposure, both size levels (thread drifters and sedentary settlers) showed a marked reduction in activity at salinities below 15, with continuous valve closure and almost complete inhibition of R and ER (Fig. 1). At salinities >15 physiological rates progressively increased, although thread drifters and sedentary settlers showed some differences (Fig. 1).

For thread drifters CR was resumed at salinity 15 and it increased almost linearly with salinity until it reached a plateau at salinity 25 (Fig. 1A). Sedentary settlers reached higher CR values than drifters once pumping was resumed (Table 1; Fig. 1A,B), but the relationship between CR and salinity showed an on/off response with a feeding activation threshold at salinity 20 (Fig. 1B). Both size and salinity were significant but so was the interaction term, probably due to the differences in the shapes of the response curves.

**Table 1:** Structure of the General Additive Models selected to describe the effect of Salinity, Size and Salinity x Size when their interaction was significant on CR, ER, R and ON during acute (2 days) and acclimated (7 days) response.

*Acute response*	Parametric Coefficient					Smooth terms (non parametrics)				R^2^ adj.	% Deviance explained	Log-likelihood Interaction		
	Parameter	Estimate	S.E.	t	P	Parameter	e.d.f.	F	P			Dif. Res. d.f.	Dif. Res. Dev.	P
*CR*	Intercept	0.132	0.008	24.48	**<2 × 10** ^**−16**^	Salinity: Drifters	3.52	15.89	**3.84 × 10** ^**−7**^	0.94	96.10%	6.63	0.0890	**8.42 × 10** ^**−15**^
	Size (Sedentary)	0.073	0.012	−6.17	**4.74 × 10** ^**−6**^	Salinity: Sedentary	6.27	56.47	** < 2 × 10** ^**−16**^					
*ER*	Intercept	0.004	0	19.83	**2.41 × 10** ^**−13**^	Salinity: Drifters	5.84	11.21	**8.61 × 10** ^**−6**^	0.91	94.60%	6.07	0.0001	** < 2 × 10** ^**−16**^
	Size (Sedentary)	0.001	0	**−**2.72	**0.014**	Salinity: Sedentary	5.85	37.59	** < 2 × 10** ^**−16**^					
*R*	Intercept	0.14	0.006	16.74	**1.06 × 10** ^**−14**^	Salinity	3.73	49.48	** < 2 × 10** ^**−16**^	0.87	89.00%	3.78	0.003	0.222
	Size (Sedentary)	**−**0.037	0.008	4.23	**2.52 × 10** ^**−4**^									
*O:N*	Intercept	28.936	2.166	7.71	**2.7 × 10** ^**−8**^	Salinity:	1	20.77	**8.5 × 10** ^**−5**^	0.55	58.00%	3.14	484.44	0.051
	Size (Sedentary)	**−**12.233	3.063	3.99	**4.49 × 10** ^**−4**^									
*CR*	Intercept	0.255	0.014	19.94	**1.03 × 10** ^**−11**^	Salinity: Drifters	1	2.37	0.146	0.77	83.30%	1.03	0.0430	**2.65 × 10** ^**−6**^
	Size (Sedentary)	0.023	0.02	**−**1.15	0.269	Salinity:Sedentary	2.93	22.47	**1.75 × 10** ^**−6**^					
*ER*	Intercept	0.003	0	19.58	**1.95 × 10** ^**−12**^	Salinity: Drifters	1.33	0.36	0.537	0.87	89.00%	1.59	2.86 × 10^**−**5^	**3.08 × 10** ^**−6**^
	Size (Sedentary)	0.004	0	**−**8.16	**4.96 × 10** ^**−7**^	Salinity:Sedentary	1	58.37	**1.05 × 10** ^**−7**^					
*R*	Intercept	0.177	0.005	25.57	**5.01 × 10** ^**−13**^	Salinity: Drifters	2.17	3.15	0.05	0.72	79.50%	2.96	0.004	**0.004**
	Size (Sedentary)	**−**0.037	0.008	4.75	**3.25 × 10** ^**−4**^	Salinity: Sedentary	2.02	7.79	**3.75 × 10** ^**−3**^					
*O:N*	Intercept	53.79	3.401	6.62	**7.84 × 10** ^**−6**^	Salinity	1.87	5.8	**0.01**	0.72	76.50%	1.69	94.62	0.593
	Size (Sedentary)	**−**31.289	4.956	6.31	**1.33 × 10** ^**−5**^									

edf: estimated degrees of freedom for the smooth terms. Dif Res. d.f.: Difference on the residual degrees of freedom between models with/without interaction. Dif. Res. Dev.: Difference on the residual deviance between models with/without interaction. Values in bold indicate statistical significance.

With regard to R, the interaction term was not significant but the size and salinity terms were (Table 1; Fig. 1C,D), indicating higher metabolic rates for thread drifters. Oxygen consumption progressively increased with salinity attaining maximum R for both size classes at salinities 20–25 to slightly decrease again at higher salinity treatments (Table 1; Fig. 1C,D).

ER relationship with salinity also differed between size classes (Table 1: significant interaction term as well as size and salinity terms) peaking at different salinities for thread drifters and sedentary settlers with maximum ammonium excretion at salinity 15 and 25, respectively (Fig. 1E,F).

Thread drifters showed higher O:N values than sedentary settlers according with the higher R and lower ER observed (Table 1). For both size classes, a linear relationship was observed between O:N and salinity, reaching maximum values at the highest salinities (30–35; Fig. 1G,H).

### Acclimated Response

Four days after the beginning of the experiment, all the treatments at salinities below 15 suffered 100% mortality in both size levels (thread drifters and sedentary settlers). Therefore, acclimated response (7^th^ day of the experiment) was only measured for salinities >15.

**Figure 2:**
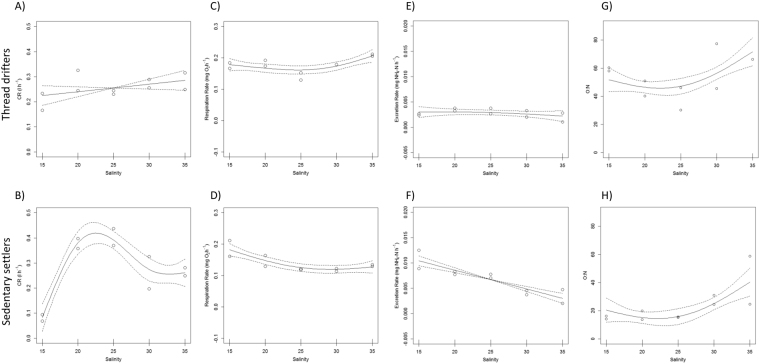
Generalized additive model results showing the partial effect of Salinity on clearance (**A**,**B**) respiration (**C**,**D**) and excretion rates (**E**,**F**) as well as O:N ratios (**G**,**H**) during acclimated response (7 days of exposure) for thread drifters (top row) and sedentary settlers (bottom row). otted lines indicate 95% confidence intervals, and tick marks along the *x*-axis below each curve represent effect values where observations occurred.

During the acclimated response, thread drifters and sedentary settlers showed again different physiological responses with regard to salinity. For thread drifters no significant relationships between salinity and any of the physiological rates (CR, R and ER) were detected (Table 1; Fig. 2A,C,E), while the physiological responses of sedentary settlers were significant for CR, R and ER (Table 1; Fig. 2B,D,F). This contrasting response resulted in significant interaction terms for Salinity and Size for CR, R, and ER (Table 1).

CR of sedentary settlers was almost completely suppressed at salinity 15, while maximum values were recorded at intermediate salinities (20–25; Fig. 2B), reinforcing the presence of a feeding activation threshold at salinity 20. R and ER showed an inverse relationship with salinity (Fig. 2D,F), although oxygen consumption reached a plateau at salinity 25 while excretion continued decreasing following a linear relationship with salinity (e.d.f = 1; Table 1).

Thread drifters continued to maintain higher O:N values than sedentary settlers, according to the higher R and lower ER recorded (Table 1). Nonetheless, both size classes showed a similar pattern between O:N and salinity with maximum values again at the higher salinity treatments (Table 1; Fig. 2G,H).

## Discussion

Most bivalve post-larvae retain a functional byssal apparatus after settlement which contributes to migrations and habitat selection^54,55^. Nonetheless, thread drifters are not usually considered as a distinct developmental stage and their contribution to shaping the distribution and abundance of populations is often downplayed, even when drifters and sedentary settlers occupy different niches. Asymmetric competition processes (inter and intra-specific) between recently settled juveniles and adults can justify the use of different habitats, but stage-specific environmental requirements may also play a role^56^. Our results point out the different physiological responses to saline stress between thread drifters and sedentary settlers both during the acute and acclimated responses (Figs 1 and 2; Table 1) highlighting the loss of drifting capability as a key developmental step with physiological implications. Thread drifters showed a higher plasticity to adapt to salinity variations, with faster acclimation processes which allowed them to maintain a more balanced metabolism (O:N ratios) over a larger range of salinities than sedentary settlers (Fig. 1; Table 1). This happened not just during the acute response as we predicted, but also during the acclimated reaction to salinity perturbation (Fig. 2; Table 1). After 7 days of exposure thread drifters were acclimated to the different salinity treatments, showing no differences in CR, R or ER between treatments as well as a balanced metabolism between proteins and carbohydrates (O:N values ≈50) for every salinity (Fig. 2). Conversely, sedentary settlers still did not resume feeding activity at salinity 15, and energy losses through respiration and excretion were larger for the lowest salinities with predomination of protein catabolism (O:N < 30) in all treatments but 35 (Fig. 2). Contrary to what we had predicted, the fact of occupying a more stable environment implies a delay of acclimation processes. Acclimation time varies between species according to their physiological limits and the degree of the salinity drop experienced^6^, but in many invertebrates also according to the developmental stage exposed to the stress episode^28,32–36^. Our results suggest that *C. edule* narrows its salinity range as they grow according to their new habitat demands. *C. edule* shows a salinity optimum around 30–35 during their planktonic larval stage while they develop in the water column^57^, but adults physiological performance maximizes at salinities 20–25^25^, consistent with the intermediate estuarine level they preferentially occupy^44^. Our results reinforce the idea of thread drifters acting as a transitional developmental stage with intermediate requirements adapted to the more variable environment they occupy. Recruitment areas are usually well defined and located towards the inner parts of the bays or estuaries at the high to mid-intertidal level while older individuals, the sedentary settlers, tend to be more abundant towards the low-intertidal and subtidal^13,44,58^. The differential distribution of age classes has been often associated with a lower predation pressure on the high-mid intertidal until spat reach certain size refuge both from adult cannibalism (≈0.9 mm^39^) and from other common predators which fed preferentially in small cockles (i.e. ≈2 mm for *Crangon crangon*^59^; 5–10 mm for *Carcinus maenas*^38^). Nonetheless, the lower predation pressure in the high intertidal is accompanied by higher variability in temperature, salinity and air exposure conditions which might explain the larger physiological plasticity of thread drifters. Higher instability has higher energetic demands with their detrimental effects on growth and fitness^6^. Drifting capability of the small spat allows them to migrate towards the low intertidal, which will be a more favourable and stable environment as they grow^12,38,60^. Since habitat demands increase with age, migration of entire cohorts might happen almost simultaneously explaining the age-stratification observed in the intertidal for some bivalves^41^.

Nonetheless, the lethal threshold was the same for both developmental stages (100% mortality after 4 days at salinity <15) and after 2 days of exposure at salinities below 15 both size levels reacted by closing their valves and depressing physiological activity (Fig. 1). Similarly, broader ranges of salinities have been reported for other bivalves such as *Mytilus charruana* spat (3–19 mm) (2–40) versus those of larger size (50–54 mm) (2–23), although the lower salinity limit was the same for both sizes^33^. Valve closure is a common behavioural response of bivalves to sudden changes in salinity which allows them to reduce salt lost from the mantle cavity fluid^4,5,25,61,62^ avoiding osmotic shock for short periods of time. During valve closure physiological activity is depressed (bradycardia, reduced respiration, etc.) and anaerobic metabolism activated^4–7^, therefore longer exposures below their lethal tolerance result in mass mortality events as has been extensively documented for cockles at salinity below 15^8,9,25^. As the lethal threshold is exceeded, acclimation processes are triggered. Avoidance of osmotic shock and oxidative stress are energetically expensive, and are usually accompanied by reductions of feeding activity and energy acquisition^4–7,5^. Feeding activity was resumed for drifters after 2 days of exposure at salinity 15 and progressively increased with salinity (Fig. 1A) indicating the activation of the acclimation processes at day 2. However, sedentary settlers kept CR close to zero at salinity 15 even after 7 days of exposure (Figs 1B and 2B) sustaining an on/off response instead of acclimation. Reductions in feeding activity during acclimation to hyposmotic environments have been reported for many bivalves species^6,29,63–68^ and might be ruled by the effect of salinity on the degree of shell gaping and siphon retraction^62^. On/off responses have been also reported in several studies on filter feeding bivalves exposed to stress conditions and described as an energy saving strategy^6,69,70^. Acclimation processes are energetically expensive, and sedentary settlers seem to be able to wait for longer before its activation.

Another indicator of activation of acclimation processes to hypo-saline stress is the increase in ammonium excretion since cell volume regulation is mainly achieved by modulation of nitrogen metabolism^5,6,26,61,71,72^. Therefore, maximum ERs might be expected at those salinities that require higher osmorregulatory effort^5,71,72^. That is the case for thread drifters during acute response which recorded maximum ER at salinity 15 (Fig. 1E). Nonetheless, sedentary settlers presented maximum ER at salinity 25 (Fig. 1F) which is the optimum salinity reported for adults of the species^25^ and also the salinity treatment which recorded maximum values of oxygen uptake (Fig. 1D) suggesting again that acclimation processes were not initiated for sedentary settlers at the lower salinities until day 7 when ER peaked at salinity 15 (Fig. 2F). Nonetheless, sedentary settlers showed greater averaged ER values than thread drifters (Table 1) in concordance with the expected increment in protein catabolism with size^73^. Allometric coefficients also differ between developmental stages with regard to respiration^74^ explaining why averaged R values were lower for sedentary settlers than thread drifters (Table 1). Higher R jointly with lower ER values explain the higher O:N ratios recorded for thread drifters (Table 1) and highlight the larger level of stress suffered by sedentary settlers to acute saline fluctuations and their lower capability for long-term acclimation.

Smaller individuals constitute the fastest-growing phase with larger demands of energy^75,76^ and therefore higher oxygen demand. Differences on metabolic activity between developmental stages might also contribute to explain their response to acclimation to saline fluctuations. Sedentary settlers might be able to slow down their metabolic activity for longer periods before the activation of the costly acclimation processes.

Physiological differences when facing saline stress might be closely related to the particular habitat occupied by each developmental stage. In more stable habitats, like the ones inhabited by sedentary settlers, where salinity drops are less common and do not last for long periods, holding physiological tuning for longer periods might compensate energetically the initiation of acclimation processes. On the other hand, highly variable habitats occupied by thread drifters might require a more plastic physiological response accompanied by a larger metabolic activity to sustain the energetic demands of continuous acclimation to the environment.

Biotic and abiotic factors interact delimiting the environmental niches of each developmental stage (inter and intra-specific competition, hydrodynamics, submersion time etc.^56^) but salinity is one of the main drivers of the distribution of the cockle populations^44,45^. Our results, point out the relevance of the thread drifters as an intermediate phase between larvae and adults. Stage-specific requirements need to be taken into account to understand population dynamics and develop integrative management strategies which integrate the spatio-temporal requirements of the species. For example, in the Schelde estuary, certain mesohaline areas are important for *C. edule* settlement during summer, but juveniles cannot survive the winter there because of the drop of salinity in that region^77^. Thus, timing of recruitment is also essential, since recently settled individuals need to grow and migrate to more suitable areas before the rainy season begins^78^. Increased variability of the climatology is already impacting the *C. edule* reproduction cycle by prolonging spawning periods and reducing resting times^20^. Recruitment timing has been described as the most important variable determining recruitment success in *C. edule* populations^27^. Changes in the gametogenetic cycle may cause mismatches between the presence of larvae, thread drifters or juveniles and their specific environmental requirements^20,78^. Complex biotic and abiotic interactions determine the optimum reproductive window of the species and the spatio-temporal habitat availability for each developmental stage. Introducing stage-specific physiological requirements on management policies is crucial to develop successful preservation strategies for the stock and the fishery in order to face new climate challenges.
